# Mental health trajectories of children and adolescents up to 5 years after the onset of the COVID-19 pandemic: a longitudinal study

**DOI:** 10.1186/s13034-026-01121-5

**Published:** 2026-06-22

**Authors:** Viviane Richard, Elsa Lorthe, Roxane Dumont, Nicolas Bovio, Natalia B. Fernandez, Mayssam Nehme, Rémy P. Barbe, Klara M. Posfay-Barbe, Idris Guessous, Silvia Stringhini, SEROCoV-KIDS study group, Andrew S. Azman, Antoine Bal, Rémy P. Barbe, Hélène Baysson, Aminata R. Bouhet, Nicolas Bovio, Paola D’Ippolito, Roxane Dumont, Nacira El Merjani, Natalia B. Fernandez, Natalie Francioli, Idris Guessous, Séverine Harnal, Julien Lamour, Arnaud G. L’Huillier, Andrea Loizeau, Elsa Lorthe, Chantal Martinez, Shannon Mechoullam, Mayssam Nehme, Klara M. Posfay-Barbe, Géraldine Poulain, Caroline Pugin, Nick Pullen, Viviane Richard, Deborah Rochat, Khadija Samir, Stephanie Schrempft, Silvia Stringhini, Stéphanie Testini, Deborah Urrutia Rivas, Anshu Uppal, Charlotte Verolet, Jennifer Villers, Guillemette Violot, María-Eugenia Zaballa

**Affiliations:** 1 Division of Primary Care Medicine, Geneva University Hospitals, Gabrielle-Perret-Gentil 4, Geneva, 1205 Switzerland; 2 Department of Health and Community Medicine, Faculty of Medicine, University of Geneva, Michel-Servet 1, Geneva, 1206 Switzerland; 3 Université Paris Cité, Inserm, INRAE, Centre for Research in Epidemiology and Statistics Paris (CRESS), Parvis Notre-Dame 1, 75004 Paris, France; 4 Geneva School of Health Sciences, HES-SO University of Applied Sciences and Arts Western Switzerland, Champel 47, Geneva, 1206 Switzerland; 5 Division of Child and Adolescent Psychiatry, Department of Woman, Child, and Adolescent Medicine, Geneva University Hospitals, Gabrielle-Perret-Gentil 4, Geneva, 1205 Switzerland; 6 Department of Pediatrics, Gynecology & Obstetrics, Pediatric Infectious Disease Unit, Faculty of Medicine, Geneva University Hospitals, Gabrielle-Perret-Gentil 4, Geneva, 1205 Switzerland; 7 School of Population and Public Health and Edwin S.H. Leong Centre for Healthy Aging, Faculty of Medicine, University of British Columbia, 117- 2194 Health Sciences Mall, Vancouver, Canada

**Keywords:** Mental health, Children, COVID-19 pandemic, Cohort, Socio-economic

## Abstract

**Background:**

The COVID-19 pandemic had heterogeneous effects on the mental health of children and adolescents according to individual experiences, with some consequences persisting beyond the lifting of restrictions. We aimed to examine whether the perceived impact of the COVID-19 pandemic was associated with 2022–2025 trajectories of mental health difficulties in children and adolescents, and to identify associated risk and protective factors.

**Methods:**

Data was drawn from the population-based SEROCoV-KIDS cohort study conducted in Geneva, Switzerland. The multidimensional perceived impact of the pandemic, as well as potential socio-demographic, health, family, social, and behavioral risk and protective factors were parent-reported at baseline, in 2022. Mental health difficulties were collected annually between 2022 and 2025. Generalized mixed effects models were used to estimate mental health trajectories by pandemic impact, and to assess risk and protective factors.

**Results:**

Of 1907 children aged 2–17 years, 9.3% and 7.9% had experienced a negative and positive pandemic impact, respectively, while most of them were not or minimally affected (82.8%). Compared to their unaffected peers, negatively impacted children had more mental health difficulties in 2022 (incidence rate ratio [IRR]: 1.51; 95% confidence interval [CI] 1.34–1.70) and improving trends between 2022 and 2025 (IRR: 0.98; 95% CI 0.95–1.01). An average-to-poor financial situation was related to a milder mental health response to a negative impact in 2022 (IRR: 0.64; 95% CI 0.46–0.89). A positive pandemic impact tended to be associated with higher difficulties in 7–12 year old children in 2022 (IRR: 1.36; 95% CI 0.98–1.89), with stable trends over time.

**Conclusion:**

About 5 years after the onset of COVID-19, the lasting mental health difficulties presented by children with a negative perceived impact of the pandemic had largely improved. Although globally reassuring, these findings call for proactive measures to prevent such long-term consequences on youth mental health in the event of future crises.

**Supplementary Information:**

The online version contains supplementary material available at https://doi.org/10.1186/s13034-026-01121-5.

## Background

The COVID-19 pandemic and the public health restrictions implemented to curb its spread affected children and adolescents worldwide [1–4]. School closures, cancellation of extracurricular activities and limitation in gatherings heavily impacted their daily life, while also representing an opportunity to spend more time with family [1, 2]. Additionally, family-level challenges, such as occupational and childcare disruptions or severe infection among relatives, had the potential to spill over into family and child functioning [1, 2]. Both negative and positive pandemic-related impacts were thus observed, with their extent varying according to children’s circumstances. In a population-based cross-sectional study conducted in Geneva, stressors, such as disturbed routines, impaired family dynamics, and decline in the financial situation were more common in children with disadvantaged economic backgrounds and unfavorable family environments [3]. Moreover, a United States study based on a convenience sample found that families with pre-existing resources, such as parental psychosocial strengths, low financial anxiety and high partner satisfaction, experienced better child and parental well-being during the pandemic [5].

In parallel to the widespread pandemic-related disruptions occurring in 2020, a global increase in mental health problems was observed in youth [6, 7]. Elevated difficulties persisted throughout 2021, despite progressive easing of measures [8–10]. Although improvements have been observed since then, mental health levels reported in October 2024 in Germany remained worse than pre-pandemic estimates [11]. Beyond these average trends, significant heterogeneity was observed in how youths’ mental health changed in response to the pandemic, reflecting diverse individual experiences [4, 12]. Negative impacts of the pandemic, such as disrupted routines, impaired family relationships, and fear for loved ones, were strongly associated with lower life satisfaction and mental health during the spring 2020 lockdown [4, 13] and up to two years after the onset of the pandemic [3]. On the other hand, positive impacts, including increased time spent with family or doing recreational activities, were related to better functioning and mental health [4].

The consequences of negative and positive pandemic impacts of the pandemic on children’s mental health could be modified by various factors. Girls and older children were more likely to display internalizing symptoms during the pandemic, suggesting a role for developmental stage, while boys presented increased externalizing difficulties [7, 14–16]. The role of socio-economic circumstances was unclear. An extensive scoping review of longitudinal or repeated cross-sectional research published between December 2019 and December 2021 found that children from lower socio-economic background were at higher risk of decreased mental health during the pandemic [17], which could be explained by greater exposure to pandemic-related stressors and limited financial and social resources to face them [2, 3]. On the contrary, a meta-analysis including articles with pre-pandemic measurements published between January 2020 and May 2022 showed that children from higher-income backgrounds experienced greater increases in depression and anxiety symptoms, which was possibly related to higher unmet social expectations because of the sanitary restrictions [7]. Additionally, resources such as family and social support were shown to be positively associated with mental health and could act as protective factors [4, 12, 18]. Similarly, a generally healthier lifestyle during the pandemic was protective of children’s well-being and mental health [19–21], while a deterioration of health behaviors, such as increased screen time and decreased physical activity, was not consistently related to decreased mental health [21, 22].

Most of these studies were conducted within the first year of the pandemic and did not assess the mid-term consequences on young people’s mental health. Furthermore, while negative effects were extensively studied [6, 7, 17], potential positive impacts of the pandemic remain poorly investigated. Finally, within the studies investigating risk and protective factors of mental health beyond the first year of the pandemic, only a few distinguished between general baseline determinants of mental health and determinants of changes during the pandemic. Among those, Zoellner et al. [23] drew on data from the German COPSY cohort collected at five timepoints between May 2020 and October 2022. They found that male sex, younger age, single parenthood, lower parental education, parental depressive symptoms and higher parental burden due to the pandemic were cross-sectionally related to lower overall child mental health at baseline, as measured with the Strengths and Difficulties questionnaire, while personal psychosocial resources and family cohesion were positively associated. Younger boys living with a single parent were more likely to experience adverse mental health changes over the follow-up period, whereas increased family cohesion and social support were related to positive changes. These results highlight the importance of longitudinal studies evaluating mental health trajectories of children following the pandemic, as well as associated risk and protective factors.

The goals of the current study were therefore to: (1) examine mid-term trajectories of children’s mental health difficulties in relation to both negative and positive impacts of the COVID-19 pandemic in Geneva, Switzerland, and (2) explore how the association between pandemic impact and mental health varied by socio-demographic, health, family, social, and behavioral factors, both cross-sectionally and longitudinally.

## Methods

### Study design and setting

Data was drawn from the SEROCoV-KIDS population-based prospective cohort study designed to assess the direct and indirect health-related consequences of the COVID-19 pandemic on children and adolescents in Geneva, Switzerland. Inclusion criteria were to be aged between 6 months and 17 years at enrollment and to live in the canton of Geneva. Children and their siblings were selected either via random sampling from state registries, or from households participating in one of the previous population-based serosurveys conducted by our group [24–27].

Baseline assessment took place between December 2021 and June 2022. One parent or referent adult per family filled out a socio-demographic, health and lifestyle questionnaire for each of their participating children, as well as one for themselves and their household. Annual follow-up surveys repeating questions about health were conducted in May-July 2023, 2024, and 2025 with an average of 16.1 (standard deviation [SD]: 2.3), 29.6 (SD: 2.4), and 39.6 months (SD: 2.2) between baseline and follow-up survey completion, respectively. Additionally, thematic questionnaires were distributed in fall 2022 and spring 2024. The Geneva Cantonal Commission for Research Ethics approved the study (ID: 2021 − 01973). All referent adults, as well as adolescents aged 14 years or older provided written consent to participate; children gave oral assent.

For the current analysis, we selected all participants aged between 2 and 17 years at enrollment and with at least one available mental health assessment. Three children with a sex reported as other were excluded because models could not be estimated with such a small category. A total of 1907 children were included in the analysis.

### Measures

#### COVID-19 pandemic impact

The impact scale of the COVID-19 Exposure and Family Impact Scales (CEFIS) was used to assess the extent to which the pandemic had affected children’s and families’ functioning, as well as their physical and emotional well-being [28]. The CEFIS was validated in United States caregivers of 0–21 year old youth [28]. In our study, this measure was collected in the fall 2022 thematic questionnaire, about eight months after the baseline assessment. Despite this, it was selected as the primary exposure because, comparing to other measures, it uniquely captures both positive and negative pandemic-related impacts. Additionally, it retrospectively assessed experiences of the pandemic since 2020, including the time before baseline, thereby partially mitigating concerns regarding temporality. CEFIS questions about how the pandemic had impacted various aspects of life (e.g. family relationships, sleep, mood) were rated on a 5-point Likert scale ranging from “Made it a lot better” to “Made it a lot worse”, with the addition of a “No change” option compared to the original version [29]. Answers were averaged across the 10 items and categorized into *Positive impact* for scores smaller than or equal to one SD below the sample mean, *Negative impact* for scores greater than or equal to one SD above the sample mean, and *No or minimal impact* otherwise. Well-being items of the CEFIS were collected for both children and parents through parent-report and combined with family interactions items to construct child- and household-level impact measures, respectively (Additional Fig. 1). The child pandemic impact was used as the main exposure, while the household measure was used in sensitivity analyses.

In further sensitivity analyses, the child-level score was recalculated excluding items related to child mood and anxiety to avoid same-construct inflation. To limit subjectivity related to threshold selection, the child-level score was also categorized into positive, no or minimal, and negative impact based on quartiles (low, combined middle-low and middle-high, high, respectively). Finally, the Coronavirus Impact Scale collected at baseline was used as a complementary measure of pandemic impact [30], with scores exceeding one SD above the sample mean considered as a negative impact [3]. This scale did not assess positive impacts.

#### Mental health difficulties

Mental health difficulties were assessed with the French version of the parent-reported Strengths and Difficulties Questionnaire (SDQ), previously used in the Swiss setting [31]. The scale was administered at baseline, in 2022, and in the May-July 2023, 2024 and 2025 follow-up questionnaires [32]. Items were added up according to scoring instructions to calculate an overall score of mental health difficulties (possible range 0–40), regarded as the primary outcome, with higher values indicating increased difficulties. In secondary analyses and as recommended for population-based samples, mental health difficulties were grouped into internalizing difficulties, including depressive and anxiety symptoms, and externalizing difficulties, such as conduct problems or attention-deficit/hyperactivity disorders (ADHD) symptoms [33]. Scores (possible range 0–20) were computed by adding the emotional and peer problems subscales, and by combining the conduct problems and hyperactivity subscales, respectively [33].

#### Socio-demographic, health, family, social, and behavioral factors

Potential risk and protective factors were parent-reported at baseline, in 2022. Socio-demographic characteristics included age, sex, the highest education level among parents (*university* versus *lower than university*), and perceived household financial situation, considered as *very good* if parents reported being able to save money, *good* if they could meet their needs and cover small unexpected expenses, and *average to poor* if they could just meet their needs or had to rely on external support. Chronic condition was defined as a physical health condition or mental behavioral and neurodevelopmental disorder diagnosed by a healthcare professional and lasting over 6 months. Family and social support comprised the referent parents’ rated relationship with the child categorized into *good* and *less than good* for answers such as rather good, rather poor and poor, the referent parents’ mood (*good* versus *average to poor*), the number of close friends (*several* versus *one or none*) collected from age six, and regular participation in extracurricular activities (*yes* versus *no*) collected from age three. Child average screen time, physical activity and sleep duration were reported in hours per day. Adherence to corresponding recommendations was assessed using age-specific thresholds from the World Health Organization [34, 35], or when not available, from the Canadian 24-Hour Movement Guidelines [36].

### Statistical analysis

The mental health difficulties total score was modelled with generalized mixed effects models following a generalized Poisson distribution, using the R glmmTMB package [37], with the child pandemic impact as main exposure. A three-level model was specified with random effects at the child and household levels. The model was adjusted for confounders by including time fixed effects for age, sex, chronic condition and household financial situation (Additional Fig. 2). Time since baseline was modeled as a time varying effect with a continuous measure to account for the varying intervals between questionnaire completion across children. 2022–2025 trajectories of mental health according to the pandemic impact were estimated by adding an interaction term between the pandemic impact and time. Sensitivity analyses were conducted by replicating the main model using alternative operationalizations of pandemic impact to mitigate potential concerns regarding the choice of threshold and possible conceptual overlap with the mental health measure. This included the household-level measure of the CEFIS, the child-level measure without emotion-related items, a quartile-based categorization of the child-level measure, and the Coronavirus Impact Scale. Additional analyses examined internalizing and externalizing mental health difficulties separately, as well as age group stratification (2–6, 7–12, and 13–17 years) to explore potential age-dependent patterns. Non-linear time trends were tested by including time as a categorical variable, by survey completion.

The moderating effect of baseline socio-demographic, health, family, social, and behavioral factors in the association between the child pandemic impact and overall mental health difficulties over time was separately tested for each moderator by including a moderator × pandemic impact and a moderator × pandemic impact × time interaction terms.

Information on the pandemic impact collected in the first thematic questionnaire was available for 1529/1907 (80.2%) children, while mental health data was complete for 1325 (69.5%), 1089 (57.1%), and 971 (50.9%) children at the first, second and third follow-up, respectively. To deal with variable missingness arising from both loss to follow-up and item non-completion (mainly financial situation: 4.0%), multiple imputation was performed. Assuming missingness at random, 40 datasets were imputed by chained equations using the R mice package [38]. The imputation model relied on the above-described variables, as well as on auxiliary variables. This included additional baseline measures of pandemic impact, such as the Coronavirus Impact Scale and questions assessing lasting changes in children’s screen time, physical activity and sleep quality due to the pandemic. One-item measures of perceived physical and mental health collected in each baseline, follow-up and thematic questionnaire were also included (Additional Table 1). Binary variables were imputed using logistic regressions, numeric variables using predictive mean matching and household financial situation using polytomous logistic regression. Household clustering could not be accounted for, as the large number of small units prevented convergence of the imputation model. The missing-at-random assumption was assessed using the delta method. Assuming that children with greater mental health difficulties were more likely to have missing follow-up data, the main model was rerun after adding 1 and 2 points to the imputed SDQ scores. Models based on full information maximum likelihood were also estimated for comparison as sensitivity analysis.

Analyses were performed with R 4.4.3 [39]. GPT-4o was used to improve the readability and language. The authors reviewed and edited all content and take full responsibility for its accuracy.

## Results

A total of 1907 children from 1134 households were included. The group was equally distributed by sex; with one third of the sample comprised of adolescents aged 13–17 and close to half of the sample comprised of 7 to 12 year olds (Table 1). According to the measure of child COVID-19 pandemic impact (mean: 2.5; SD: 0.23), 9.3% of children had experienced a negative impact, mostly driven by changes in anxiety and mood (Additional Fig. 1), and 7.9% a positive impact, while most of them were considered minimally or non-affected (82.8%). A negative impact of the pandemic was more frequently reported in children with a chronic condition, unfavorable family and social situations and not adhering to health behaviors recommendations than in their counterparts (Table 1). An average-to-poor household financial situation was related to both negative and positive impacts.

Children aged 13–17 years at baseline, with poorer socio-economic circumstances and lower adherence to health behavior recommendations were more likely to have missing data, although patterns of non-response varied across questionnaires (Additional Table 2). Modelling results presented hereafter rely on analysis with imputed data for missing baseline variables and incomplete later questionnaires. Diagnostics of the main models including visual assessments of linearity, residual normality, and homoscedasticity, were deemed acceptable (Additional Fig. 3).

**Table 1:** Participants’ baseline characteristics according to the child COVID-19 pandemic impact

	Total included (*n* = 1907)	Child COVID-19 pandemic impact^e^					
		Total with impact (*n* = 1488)	None or minimal (*n* = 1233)	Negative (*n* = 138)	Positive (*n* = 117)		*p*-value
	n (%)	n (%)	n (%)	n (%)	n (%)		
Age category (*n*=1858^a^)							
2–6 years	419 (22.5)	350 (23.5)	294 (23.8)	28 (20.3)	28 (23.9)		0.173
7–12 years	873 (47.0)	735 (49.4)	609 (49.4)	62 (44.9)	64 (54.7)		
13–17 years	566 (30.5)	403 (27.1)	330 (26.8)	48 (34.8)	25 (21.4)		
Sex (*n* = 1907)							
Male	955 (50.1)	751 (50.5)	630 (51.1)	65 (47.1)	56 (47.9)		0.566
Female	952 (49.9)	737 (49.5)	603 (48.9)	73 (52.9)	61 (52.1)		
Chronic condition (*n* = 1857)							
No	1354 (70.2)	1,049 (70.5)	880 (71.4)	80 (58.0)	89 (76.1)		0.002
Yes	553 (29.8)	439 (29.5)	353 (28.6)	58 (42.0)	28 (23.9)		
Household financial situation (*n* = 1783)							
Very good	670 (37.6)	541 (38.3)	470 (40.2)	33 (25.0)	38 (34.6)		0.003
Good	782 (43.9)	635 (45.0)	520 (44.4)	68 (51.5)	47 (42.7)		
Average-to-poor	331 (18.5)	236 (16.7)	180 (15.4)	31 (23.5)	25 (22.7)		
Parents’ highest education (*n* = 1899)							
University	1,588 (83.6)	1254 (84.3)	1038 (84.2)	117 (84.8)	99 (84.6)		0.978
Lower than university	311 (16.4)	234 (15.7)	195 (15.8)	21 (15.2)	18 (15.4)		
Parent-child relationship (*n* = 1858)							
Good	1525 (82.1)	1225 (82.3)	1,036 (84.0)	90 (65.2)	99 (84.6)		< 0.001
Less than good	333 (17.9)	263 (17.7)	197 (16.0)	48 (34.8)	18 (15.4)		
Parents’ mood (*n* = 1898)							
Good	1,655 (87.2)	1,303 (87.6)	1,103 (89.5)	98 (71.0)	102 (87.2)		< 0.001
Average to poor	243 (12.8)	184 (12.4)	129 (10.5)	40 (29.0)	15 (12.8)		
Number of close friends (*n*=1528^b^)							
Several	1212 (79.3)	966 (79.1)	809 (80.2)	79 (68.1)	78 (80.4)		0.010
One or none	316 (20.7)	256 (20.9)	200 (19.8)	37 (31.9)	19 (19.6)		
Participation in extracurricular activities (*n*=1797^*c*^)							
Yes	1566 (87.1)	1281 (88.5)	1077 (89.8)	108 (81.8)	96 (83.5)		0.005
No	231 (12.9)	166 (11.5)	123 (10.2)	24 (18.2)	19 (16.5)		
Screen time (*n* = 1811)							
Meeting recommendations	1311 (72.4)	1099 (75.6)	918 (76.2)	89 (66.9)	92 (80.0)		0.032
Not meeting recommendations	500 (27.6)	354 (24.4)	287 (23.8)	44 (33.1)	23 (20.0)		
Physical activity (*n* = 1843)							
Meeting recommendations	1241 (67.3)	1001 (67.8)	849 (69.3)	77 (56.6)	75 (64.7)		0.008
Not meeting recommendations	602 (32.7)	476 (32.2)	376 (30.7)	59 (43.4)	41 (35.3)		
Sleep duration (*n* = 1857)							
Meeting recommendations	1460 (78.6)	1193 (80.2)	999 (81.0)	96 (69.6)	98 (83.8)		0.004
Not meeting recommendations	397 (21.4)	295 (19.8)	234 (19.0)	42 (30.4)	19 (16.2)		
Mental health difficulties (*n* = 1857) ^*d*^	mean (SD)	mean (SD)	mean (SD)	mean (SD)	mean (SD)		
Overall score (sample range: 0–28)	7.1 (4.9)	7.09 (4.94)	6.59 (4.55)	11.65 (5.91)	6.97 (4.87)		< 0.001
Internalizing score (sample range: 0–17)	2.9 (2.7)	2.86 (2.66)	2.58 (2.39)	5.26 (3.67)	2.92 (2.58)		< 0.001
Externalizing score (sample range: 0–17)	4.2 (3.4)	4.23 (3.42)	4.01 (3.29)	6.39 (3.85)	4.04 (3.39)		< 0.001

p-values from chi^2^ and Kruskal-Wallis tests comparing types of pandemic impact according to categorical and continuous baseline variables, respectively. SD stands for standard deviation.

^a^Missing for children without baseline information

^b^Not collected among 322 participants < age 6.

^c^Not collected among 61 participants < age 3.

^d^Mental health difficulties assessed with the Strengths and difficulties questionnaire (SDQ), with higher values indicating heightened difficulties.

^e^Pandemic impact assessed with the COVID-19 Exposure and Family Impact Scale (CEFIS) in a survey with 1529/1907 (80.2%) response rate and categorized with the sample’s mean +/**-** 1 SD

### Mental health difficulties according to the pandemic impact at baseline and over time

**Fig. 1:**
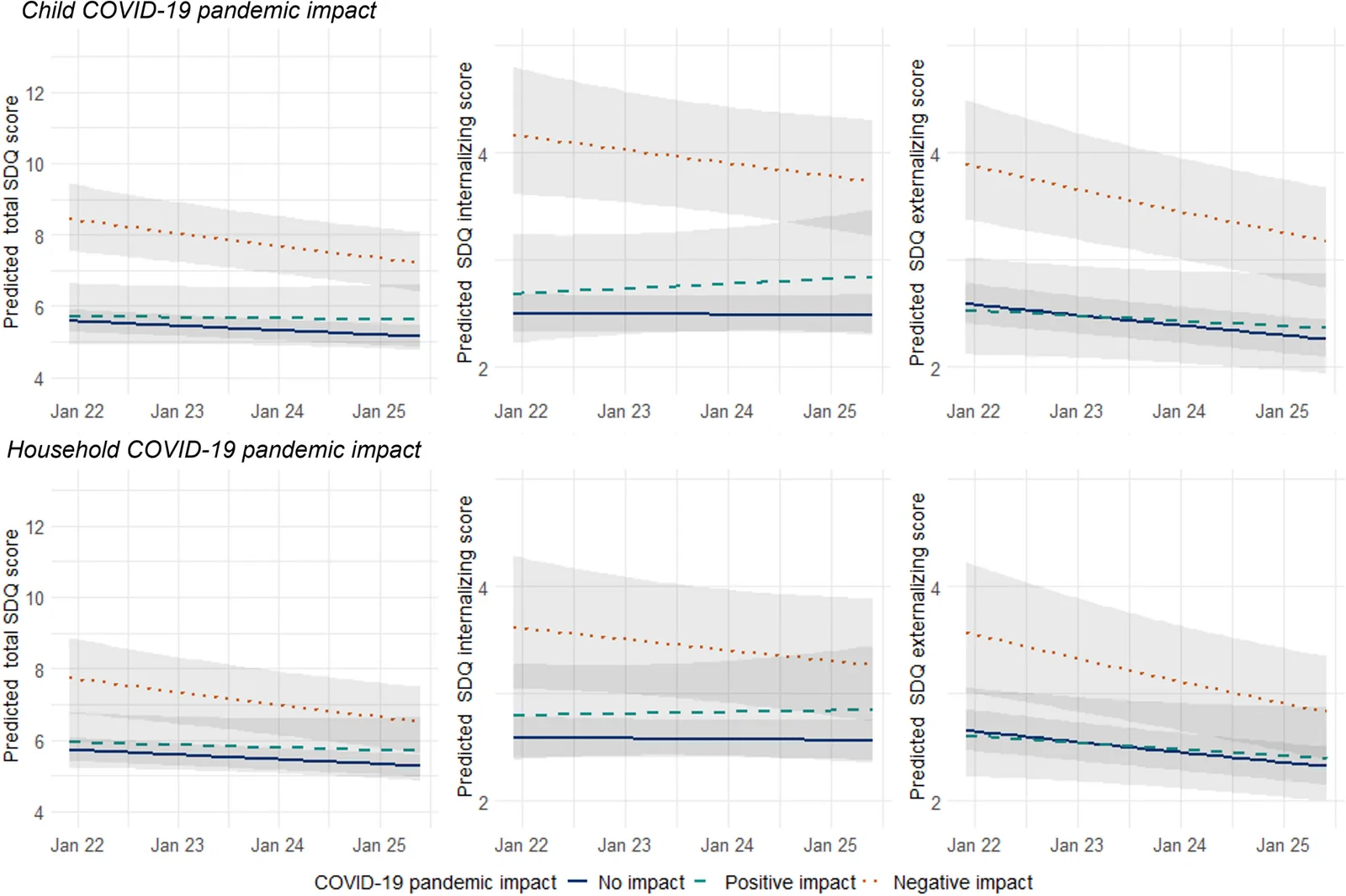
Trajectories of mental health difficulties according to the child and household impact of the COVID-19 pandemic in children aged 2–17 years, between 2022 and 2025 (*n* = 1907), with higher strengths and difficulties questionnaire (SDQ) scores indicating heightened difficulties. Marginal score prediction and 95% confidence interval (shaded area) from generalized mixed effects models adjusted with fixed effects for age, sex, chronic condition, and household financial situation, and random effects at the child and household levels. Missing values imputed with multiple imputations by chained equations

At baseline, in 2022, participants who had experienced a negative pandemic impact had 51% higher scores of mental health difficulties (incidence rate ratio [IRR]: 1.51; 95% confidence interval [CI]: 1.34–1.70) than those experiencing no or minimal impact, resulting in a score difference of 3.1 points (Fig. 1). There was no significant mental health difference between experiencing a positive and no pandemic impact (p-value > 0.1).

Compared to children with no or minimal reported impact, those negatively impacted by the pandemic showed a non-significant annual decrease of 2% (IRR: 0.98; 95% CI: 0.95–1.01) in the overall mental health difficulties score between 2022 and 2025 (Fig. 1 & Additional Table 3). However, when restricting the analysis to the period between 2022 and 2024, this downward trend became steeper and statistically significant (IRR: 0.96; 95% CI: 0.92–0.99), as mental health difficulties slightly increased again in 2025 among negatively impacted children (Additional Fig. 4). This was also observed when treating time as a categorical variable, with fewer mental health difficulties reported among negatively impacted children in 2024 than in 2022 (IRR: 0.88; 95% CI: 0.79–0.98), while the difference was smaller and not significant in 2025 (Additional Table 4). The evolution of mental health did not differ between not impacted and positively impacted children (Fig. 1 & Additional Table 3). Similar trends were observed when relying on different definitions of pandemic impact (Fig. 1 & Additional Tables 3 and 5). Furthermore, these patterns were consistent across internalizing and externalizing mental health difficulties and across age groups, although not significantly (Fig. 1 & Additional Table 3).

### Risk and protective factors at baseline and over time

**Fig. 2:**
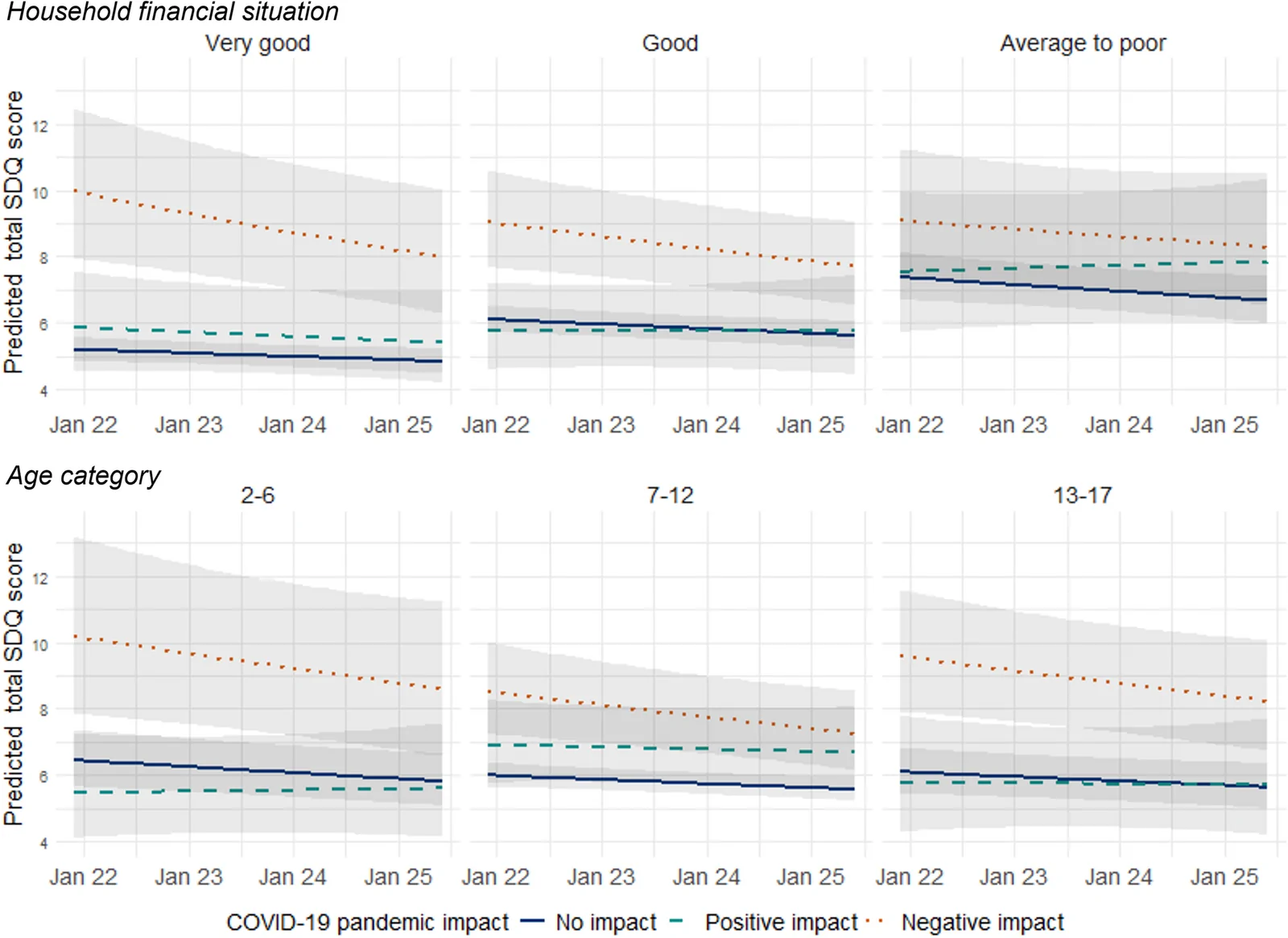
Trajectories of mental health difficulties according to the child impact of the COVID-19 pandemic, household financial situation and age category in children aged 2–17 years, between 2022 and 2025 (*n* = 1907), with higher strengths and difficulties questionnaire (SDQ) scores indicating heightened difficulties. Marginal score prediction and 95% confidence interval (shaded area) from generalized mixed effects model adjusted with fixed effects for age, sex, chronic condition, and household financial situation, and random effects at the child and household levels. Missing values imputed with multiple imputations by chained equations

When exploring potential moderators, the baseline association between a negative pandemic impact and elevated mental health difficulties was weaker in children with an average-to-poor (IRR: 0.64; 95% CI: 0.46–0.89) or good financial situation (IRR: 0.77; 95% CI: 0.57–1.04) than in their most privileged counterparts (Fig. 2 & Additional Table 6). Specifically, at baseline, the difference in SDQ score between negatively and non-impacted children was 6.1 points in those with a very good financial situation, while it was 1.9 points in those with an average to poor situation, representing a 4.2 points smaller difference. However, living in a household with an average-to-poor financial situation (IRR: 1.42; 95% CI: 1.26–1.60), and to a lesser extent a good financial situation (IRR: 1.18; 95% CI: 1.07–1.29), was related to higher average levels of mental health difficulties (Fig. 2). A positive pandemic impact tended to be associated with higher baseline mental health difficulties in 7–12 year old children than in younger ones (IRR: 1.36; 95% CI: 0.98–1.89; Fig. 2, Additional Table 6). There was no other moderator affecting the baseline pandemic impact on mental health difficulties (Additional Table 6).

Over time, the rate of change in mental health difficulties according to the pandemic impact did not vary according to any moderator under study (Additional Table 6). Analyses with maximum likelihood revealed similar patterns and effect sizes as those conducted with imputed data (Additional Tables 3 and 6). Models based on imputed data using the delta method under the assumption that children with missing follow-up were more likely to have mental health difficulties yielded similar, and even more pronounced, estimates (Additional Table 7).

## Discussion

In 2022, two years after the onset of COVID-19, children who had experienced a negative impact of the pandemic had higher mental health difficulties than their non-affected counterparts, particularly those with a privileged financial situation. From 2022 to 2024, mental health difficulties tended to subside in negatively impacted children, before slightly increasing again in 2025. Positively impacted 7–12 year old children seemed to have more baseline mental health difficulties than their non-impacted peers and followed a stable trend over time, while there was no difference in younger or older children.

Four out of five children did not seem meaningfully impacted by the pandemic and displayed a good mental health over the 2022–2025 period, with low levels of anxiety and depressive symptoms (internalizing difficulties) or conduct problems and ADHD symptoms (externalizing difficulties). It echoes evidence from Germany, where 64–74% of children followed a stable low trajectory of mental health problems between 2020 and 2022 and constituted the group least burdened by the pandemic [12]. Conversely and as previously observed [4, 13], children who were negatively impacted by the pandemic displayed poorer mental health than their unaffected counterparts. Reflecting previous national and international findings [3, 11, 14], this association was noticeable two years into the pandemic, even after most restrictions had been lifted. We estimated the difference to be of 3.1 SDQ points in early 2022, with each additional point corresponding to a 1.28-fold increase in the odds of presenting a mental health disorder [40]. These results are specific to the Swiss context and given that Switzerland implemented relatively light pandemic-related restrictions (e.g. no inland travel restriction, school closure limited to 10 weeks in Spring 2020), they might represent a lower burden than in countries with stricter measures [41]. It also probably constituted an already improved situation compared to the first year of the pandemic [11]. Reassuringly, by summer 2024, the mental health of negatively impacted children seemed to have improved although it remained somewhat poorer than the levels displayed by non-affected children, suggesting lingering consequences of the pandemic. This is in line with a German study showing that the high levels of mental health difficulties observed in 2020 improved in the following years but were still slightly higher in October 2024 than before the pandemic [11]. In Icelandic adolescents depressive symptoms had decreased in 2023 compared to 2021 but remained higher than before the pandemic, while anxiety and hostility showed little improvement by 2023 [42]. We observed a slight rebound in difficulties among negatively impacted children in summer 2025. This rebound is unlikely to be attributable to the COVID-19 pandemic, given the absence of an active outbreak and the lifting of pandemic-related measures in February 2022 [43]. However, these children may be particularly sensitive to external stressors and could have been affected by other events. The modest and non-significant rebound might also reflect random fluctuation in the sample.

An unfavorable financial situation was associated with poorer levels of mental health on average but was related to milder mental health consequences following a negative pandemic impact. The magnitude of this absolute difference (4.2 SDQ points) suggests that the buffering effect may be clinically relevant. The observed social gradient in mental health was in line with the extensive literature on health inequalities [44]. Pandemic-related patterns echoed a meta-analysis and a Swiss study reporting that children with higher socio-economic circumstances experienced a greater decrease in well-being during the pandemic [7, 45], but contradicted results from an extensive scoping review [17]. A potential explanation for our finding is that a negative pandemic impact was perceived as a comparatively lesser burden by disadvantaged children who are more familiar with adverse circumstances and could have developed coping strategies to deal with them, than by advantaged children who might have had more unmet expectations [7, 9, 46]. It could also be that a negative pandemic impact was related to a certain level of heightened mental health difficulties, similar across all children, that represented a greater increase for privileged individuals who had better average levels than their less advantaged counterparts.

A positive pandemic impact tended to be persistently associated with elevated mental health difficulties between 2022 and 2025 in 7-12-year old children but not in younger children and adolescents. It may be that primary school-aged children and their parents who enjoyed a relieved situation during the lockdown period found it difficult to return to a normal routine after experiencing an improved situation [46]. For instance, a tentative interpretation might be that children with learning disorders or social anxiety benefited from following their own rhythm and being away from daily stressors, including onsite assignment and peer interactions [47]. The onset of such conditions typically occurs from the start of school [48, 49], which could explain why a positive pandemic impact was associated with elevated problems between 2022 and 2025 in 7–12 but not 2–6 year old children. In positively impacted adolescents, potential difficulties related to the return to normalcy might have been outweighed by their developmental need for interactions and experiences outside of the family environment [50], hence the absence of difference in this age group. Neurodevelopmental stage, which is known to play an important role in the onset of mental health disorders, might also explain the different response of 7–12 year old children compared to other age groups [51].

Even if the improving trend is reassuring, children with the heaviest pandemic burden faced higher levels of mental health difficulties up to five years after the onset of the pandemic and may represent a group particularly vulnerable to other major societal events. This highlights the need to consider the psychological well-being of young populations in public responses to crises. Specifically, a consistent routine and age-adapted parent-child communication were shown to be effective in buffering the impact of the pandemic and could be applied to other situations [52]. Since such mitigation approaches rely on the involvement and availability of parents, tailored measures supporting them should also be considered, including flexible working arrangements, when possible, psychological counselling, and financial aid. Finally, policies could incorporate positive lessons learned from the pandemic to better address the needs of all children. For instance, possibilities of adapted education could be explored for children who benefited more from distance than onsite learning [46].

These findings should be interpreted in light of their limitations. First, although referring to the retrospective impact of the pandemic, the measure of pandemic impact was assessed approximately eight months after the baseline measurement of mental health difficulties, raising concerns regarding temporality. However, sensitivity analyses using the Coronavirus Impact Scale measured concurrently with baseline mental health, yielded similar estimates, thereby supporting the robustness of the findings. Additionally, the pandemic impact was parent-reported two years into the pandemic and was thus subject to recall and proxy biases, with the perception of the impact severity potentially varying according to personal traits [53]. Second, despite the random selection of participants, children from highly educated parents were more likely to participate, resulting in a sample overrepresenting socially advantaged individuals in comparison to the Geneva population [54] thereby limiting external validity. Therefore, results might not be generalizable to children living in precarious circumstances and might underestimate the association between socio-economic conditions and mental health. Third, despite continuous efforts to retain participants, attrition significantly increased over time. However, multiple imputations and full-information maximum likelihood approaches used in the current analysis are thought to be robust to loss to follow-up in cohort studies [55], especially since sensitivity analysis showed no evidence of missing not at random patterns. Finally, we acknowledge that mental health is shaped by a complex and dynamic interplay of multiple determinants, making it challenging to disentangle the effect of the pandemic from age and other factors related to children’s development. Our study also has major strengths including the sizeable population-based sample covering a wide age range, the longitudinal design with multiple assessments up to five years after the COVID-19 pandemic onset, the exploration of the effect of a positive pandemic impact and the inclusion of various socio-demographic, health, family, social, and behavioral moderators.

## Conclusion

About five years after the onset of the COVID-19, the lasting mental health difficulties presented by negatively impacted children, including internalizing and externalizing symptoms, had largely improved. However, these reassuring findings should not mask that youth mental health was affected for years following the pandemic and might be sensitive to other societal events. Drawing on the extensive research conducted during the pandemic, more consideration and resources should be allocated to foster children’s and adolescents’ mental health in the event of future crises.

## Supplementary Information

Below is the link to the electronic supplementary material.

Supplementary Material 1.

## Data Availability

The dataset used during the current study is available from the corresponding author on reasonable request.

## Abbreviations

CEFIS: COVID-19 exposure and family impact scales
CI: Confidence interval
COVID-19: Coronavirus disease 2019
IRR: Incidence rate ratio
SD: Standard deviation
SDQ: Strengths and difficulties questionnaire
