# Supplementary material for: Mental health trajectories of children and adolescents up to 5 years after the onset of the COVID-19 pandemic: a longitudinal study
Source: Child Adolesc Psychiatry Ment Health. 2026 Jun 22;20:126. doi: 10.1186/s13034-026-01121-5 (PMC13576257; doi:10.1186/s13034-026-01121-5)
Supplement: Supplementary file 1 [file 13034_2026_1121_MOESM1_ESM.pdf]

Mental health trajectories of children and adolescents up to five years after the onset of the COVID-19 pandemic: a longitudinal study

Additional files – Child and Adolescent Psychiatry and Mental Health

Viviane Richard, Elsa Lorthe, Roxane Dumont, Nicolas Bovio, Natalia Fernandez, Mayssam Nehme, Rémy P. Barbe, Klara M. Posfay-Barbe, Idris Guessous, Silvia Stringhini, SEROCoV-KIDS study group

Correspondence to
Silvia Stringhini
silvia.stringhini@ubc.ca
Unité d'épidémiologie populationnelle
Rue Jean-Violette 29
1205 Genève
Switzerland
+41 22 305 58 61

Additional table 1. Auxiliary measures used in multiple imputation models

Question	Timepoint
Coronavirus impact scale: How much has the pandemic changed the child's life in the following ways? [routine, food access, physical healthcare access, mental healthcare access, social network, stress level, family tensions, family income]<br>- No change<br>- Mild<br>- Moderate<br>- Severe	Baseline
In your opinion, has the pandemic had a lasting impact on the amount of time the child spends in front of a screen for recreational activities?<br>- No, this time has remained the same<br>- Yes, this time has increased<br>- Yes, this time has decreased<br>- I don't know	Baseline
In your opinion, has the pandemic had a lasting impact on the time spent doing physical exercise?<br>- No, this time has remained the same<br>- Yes, this time has increased<br>- Yes, this time has decreased<br>- I don't know	Baseline
In your opinion, has the pandemic had a lasting impact on the quality of their sleep?<br>- No, it has not changed<br>- Yes, it has improved<br>- Yes, it is worse	Baseline
In general, how would you rate the child's physical health?<br>- Very good<br>- Good<br>- Average<br>- Poor<br>- Very poor	Baseline,<br>Fall 2022,<br>Summer 2023,<br>Spring 2024,<br>Summer 2024,<br>Summer 2025
In general, how would you rate their morale?<br>- Very good<br>- Good<br>- Average<br>- Poor<br>- Very poor	Baseline,<br>Fall 2022,<br>Summer 2023,<br>Spring 2024,<br>Summer 2024,<br>Summer 2025

Additional table 2. Participants' baseline characteristics according to response to follow-up surveys.

	Total (n=1907)	Survey non-response: Thematic survey (n=378)	p-value	1st follow-up (n=585)	p-value	2nd follow-up (n=820)	p-value	3rd follow-up (n=936)	p-value
**Age category (n=1858)**									
2-6 years	419 (22.6)	54 (16.4)	<0.001	126 (22.2)	0.277	163 (20.5)	<0.001	183 (20.3)	<0.001
7-12 years	873 (47.0)	120 (36.5)		254 (44.8)		349 (43.9)		397 (44.0)	
13-17 years	566 (30.5)	155 (47.1)		187 (33.0)		283 (35.6)		322 (35.7)	
**Sex (n=1907)**									
Male	955 (50.1)	185 (48.9)	0.663	303 (51.8)	0.343	420 (51.2)	0.413	483 (51.6)	0.207
Female	952 (49.9)	193 (51.1)		282 (48.2)		400 (48.8)		453 (48.4)	
**Chronic condition (n=1857)**									
No	1304 (70.2)	225 (68.6)	0.521	410 (72.3)	0.211	540 (67.9)	0.069	621 (68.8)	0.227
Yes	553 (29.8)	103 (31.4)		157 (27.7)		255 (32.1)		281 (31.2)	
**Household financial situation (n=1783)**									
Very good	670 (37.6)	115 (34.3)	0.003	199 (37.5)	0.338	269 (36.0)	0.372	285 (33.1)	<0.001
Good	782 (43.9)	136 (40.6)		223 (42.0)		331 (44.3)		396 (45.9)	
Average-to-poor	331 (18.6)	84 (25.1)		109 (20.5)		148 (19.8)		181 (21.0)	
**Parents' highest education (n=1899)**									
University	1588 (83.6)	308 (83.2)	0.887	481 (82.4)	0.357	660 (80.9)	0.006	746 (80.3)	<0.001
Lower than university	311 (16.4)	62 (16.8)		103 (17.6)		156 (19.1)		183 (19.7)	
**Parent-child relationship (n=1858)**									
Good	1525 (82.1)	266 (80.9)	0.575	466 (82.2)	0.987	640 (80.5)	0.142	744 (82.5)	0.702
Less than good	333 (17.9)	63 (19.1)		101 (17.8)		155 (19.5)		158 (17.5)	
**Parents' mood (n=1898)**									
Good	1655 (87.2)	315 (85.1)	0.216	507 (87.0)	0.898	706 (86.5)	0.485	801 (86.2)	0.239
Average to poor	243 (12.8)	55 (14.9)		76 (13.0)		110 (13.5)		128 (13.8)	
**Number of close friends (n=1528)**									
Several	1212 (79.3)	225 (80.1)	0.793	375 (80.5)	0.504	525 (78.8)	0.724	594 (78.4)	0.394
One or less	316 (20.7)	56 (19.9)		91 (19.5)		141 (21.2)		164 (21.6)	
**Participation in extracurricular activities (n=1797)[a]**									
Yes	1566 (87.1)	264 (82.8)	0.013	469 (85.0)	0.078	645 (83.7)	<0.001	745 (84.9)	0.008
No	231 (12.9)	55 (17.2)		83 (15.0)		126 (16.3)		132 (15.1)	
**Screen time (n=1811)**									
Meeting recommendations	1311 (72.4)	184 (57.9)	<0.001	389 (71.2)	0.510	521 (67.9)	<0.001	590 (67.6)	<0.001
Not meeting recommendations	500 (27.6)	134 (42.1)		157 (28.8)		246 (32.1)		283 (32.4)	
**Physical activity (n=1843)[b]**									
Meeting recommendations	1241 (67.3)	214 (65.6)	0.514	374 (67.8)	0.845	519 (66.3)	0.437	590 (66.3)	0.382
Not meeting recommendations	602 (32.7)	112 (34.4)		178 (32.2)		264 (33.7)		300 (33.7)	
**Sleep duration (n=1857)**									
Meeting recommendations	1460 (78.6)	238 (72.3)	0.003	432 (76.2)	0.103	594 (74.7)	<0.001	679 (75.3)	0.001
Not meeting recommendations	397 (21.4)	91 (27.7)		135 (23.8)		201 (25.3)		223 (24.7)	
**Mental health difficulties total score[c]**	7.1 (4.9)	7.1 (4.9)	0.955	7.2 (4.8)	0.813	7.4 (4.9)	0.035	7.3 (4.8)	0.210
**Internalizing difficulties score[c]**	2.9 (2.7)	3.0 (2.8)	0.431	2.8 (2.5)	0.189	3.0 (2.8)	0.068	2.9 (2.7)	0.651
**Externalizing difficulties score[c]**	4.2 (3.4)	4.1 (3.4)	0.483	4.4 (3.5)	0.168	4.4 (3.3)	0.109	4.4 (3.4)	0.147

Results are numbers (%) for categorical variables and mean (standard deviation) for continuous ones. P-values compare survey non-respondents to respondents and are from Chi-squared test for categorical variables and analysis of variance for continuous ones. Baseline survey: December 2021 – June 2022; thematic survey: September 2022 – January 2023; 1st follow-up: May – July 2023; 1st follow-up: May – July 2024; 1st follow-up: May – July 2025.

[a] Not collected among 322 participants < 6 years old.

[b] Not collected among 61 participants < 3 years old.

[c] Mental health difficulties assessed with the strengths and difficulties questionnaire (SDQ), with higher values indicating heightened difficulties.

Additional table 3. Yearly incidence rate ratio of mental health difficulties according to the COVID-19 pandemic impact in children aged 2-17 years old, between 2022 and 2025 (n=1907).

COVID-19 pandemic impact measure	Type of impact	Mental health difficulties		Internalizing difficulties		Externalizing difficulties	
		Maximum likelihood	Multiple imputation	Maximum likelihood	Multiple imputation	Maximum likelihood	Multiple imputation
		IRR (95% CI)	IRR (95% CI)	IRR (95% CI)	IRR (95% CI)	IRR (95% CI)	IRR (95% CI)
Child impact	None/minimal	Ref.	Ref.	Ref.	Ref.	Ref.	Ref.
Child impact	Positive	1.03 (0.99; 1.07)	1.02 (0.98; 1.06)	1.02 (0.97; 1.09)	1.02 (0.96; 1.08)	1.03 (0.98; 1.08)	1.02 (0.98; 1.06)
Child impact	Negative	0.97 (0.94; 1.00)°	0.98 (0.95; 1.01)	0.98 (0.94; 1.02)	0.97 (0.93; 1.01)	0.97 (0.93; 1.01)°	0.98 (0.95; 1.01)
Household impact	None/minimal	Ref.	Ref.	Ref.	Ref.	Ref.	Ref.
Household impact	Positive	1.02 (0.98; 1.05)	1.01 (0.98; 1.05)	1.01 (0.96; 1.06)	1.01 (0.96; 1.06)	1.02 (0.98; 1.07)	1.01 (0.98; 1.06)
Household impact	Negative	0.97 (0.93; 1.00)°	0.97 (0.94; 1.01)	0.98 (0.93; 1.03)	0.97 (0.94; 1.01)	0.96 (0.92; 1.00)°	0.97 (0.93; 1.01)

° p-value < 0.1. Results are incidence rate ratio (IRR) and 95% confidence intervals (CI) of pandemic impact × time interactions from generalized mixed effects models with fixed effects for age, sex, chronic condition, and household financial situation, and random effects at the child and household levels.

Additional table 4. Change in mental health difficulties according to the child COVID-19 pandemic impact and survey year in children aged 2-17 years old, between 2022 and 2025

Type of child impact	Timepoint	Mental health difficulties	Internalizing difficulties	Externalizing difficulties
		IRR (95% CI)	IRR (95% CI)	IRR (95% CI)
Negative	2022	Ref.	Ref.	Ref.
	2023	0.94 (0.86-1.04)	0.94 (0.82-1.08)	0.94 (0.84-1.06)
	2024	0.88 (0.79-0.98)*	0.87 (0.74-1.01)°	0.89 (0.78-1.01)°
	2025	0.95 (0.85-1.05)	0.96 (0.83-1.12)	0.92 (0.80-1.05)
Positive	2022	Ref.	Ref.	Ref.
	2023	1.07 (0.94-1.22)	1.04 (0.86-1.26)	1.08 (0.93-1.27)
	2024	1.08 (0.93-1.25)	1.04 (0.84-1.29)	1.10 (0.93-1.31)
	2025	1.07 (0.93-1.24)	1.06 (0.86-1.31)	1.08 (0.91-1.28)

° p-value < 0.1; * < 0.05. Results are incidence rate ratio (IRR) and 95% confidence intervals (CI) of pandemic impact × time category interactions from generalized mixed effects models with fixed effects for age, sex, chronic condition, and household financial situation, and random effects at the child and household levels. Variable missingness is addressed with full-information maximum likelihood.

Additional table 5. Yearly incidence rate ratio of mental health difficulties according to the COVID-19 pandemic impact, by age category and impact measure in children aged 2-17 years old, between 2022 and 2025.

**Age category**	**COVID-19 pandemic impact measure**	**Type of impact**	**Mental health difficulties**	**Internalizing difficulties**	**Externalizing difficulties**
			IRR (95% CI)	IRR (95% CI)	IRR (95% CI)
2-6	Child impact	None/minimal	Ref.	Ref.	Ref.
2-6	Child impact	Positive	1.04 (0.96; 1.13)	1.06 (0.93; 1.22)	1.04 (0.96; 1.13)
2-6	Child impact	Negative	0.96 (0.90; 1.03)	1.02 (0.91; 1.14)	0.93 (0.86; 1.01)°
7-12	Child impact	None/minimal	Ref.	Ref.	Ref.
7-12	Child impact	Positive	1.03 (0.98; 1.09)	1.02 (0.94; 1.09)	1.04 (0.97; 1.11)
7-12	Child impact	Negative	0.97 (0.93; 1.01)	0.95 (0.89; 1.01)°	0.98 (0.93; 1.03)
13-17	Child impact	None/minimal	Ref.	Ref.	Ref.
13-17	Child impact	Positive	1.02 (0.91; 1.14)	1.04 (0.91; 1.19)	0.98 (0.84; 1.15)
13-17	Child impact	Negative	0.99 (0.94; 1.04)	0.99 (0.92; 1.06)	0.98 (0.91; 1.05)
All	Coronavirus Impact Scale	None/minimal	Ref.	Ref.	Ref.
All	Coronavirus Impact Scale	Negative	0.98 (0.95;1.00)°	0.97 (0.93;1.01)	0.98 (0.94;1.01)
All	Child impact quartiles	None/minimal	Ref.	Ref.	Ref.
All	Child impact quartiles	Positive	1.00 (0.98;1.03)	1.00 (0.96;1.05)	1.00 (0.97;1.04)
All	Child impact quartiles	Negative	0.97 (0.94;0.99)*	0.96 (0.93;1.00)*	0.96 (0.94;0.99)*
All	Child impact without emotion items	None/minimal			
All	Child impact without emotion items	Positive	1.03 (0.99-1.07)	1.03 (0.97-1.08)	1.03 (0.98-1.08)
All	Child impact without emotion items	Negative	0.97 (0.94-1.00)°	0.96 (0.92-1.01)	0.98 (0.94-1.02)

° p-value < 0.1; * < 0.05

Results are incidence rate ratio (IRR) and 95% confidence intervals (CI) of pandemic impact × time interactions from generalized mixed effects models with fixed effects for age, sex, chronic condition, and household financial situation, and random effects at the child and household levels. Variable missingness is addressed with full-information maximum likelihood.

Additional table 6. Yearly incidence rate ratio of mental health difficulties according to the child COVID-19 pandemic impact and socio-demographic, health, family, social and behavioral moderators, in children aged 2-17 years old, between 2022 and 2025 (n=1907).

Moderator	Child COVID-19 pandemic impact	Moderator × pandemic impact interaction		Moderator × pandemic impact × time interaction	
		Maximum likelihood	Multiple imputation	Maximum likelihood	Multiple imputation
		IRR (95% CI)	IRR (95% CI)	IRR (95% CI)	IRR (95% CI)
**Age category**					
2-6	None or minimal	Ref.	Ref.	Ref.	Ref.
7-12	Positive	1.47 (1.06; 2.02)*	1.36 (0.98; 1.89)°	0.99 (0.91; 1.09)	0.98 (0.89; 1.07)
7-12	Negative	0.87 (0.64; 1.19)	0.90 (0.67; 1.22)	1.01 (0.93; 1.09)	0.99 (0.92; 1.07)
13-17	Positive	1.26 (0.83; 1.92)	1.12 (0.76; 1.66)	0.98 (0.86; 1.12)	0.98 (0.88; 1.10)
13-17	Negative	0.97 (0.69; 1.35)	1.00 (0.73; 1.36)	1.03 (0.94; 1.12)	1.00 (0.92; 1.08)
**Sex**					
Male	None or minimal	Ref.	Ref.	Ref.	Ref.
Female	Positive	1.07 (0.82; 1.40)	1.08 (0.84; 1.39)	0.96 (0.89; 1.04)	0.98 (0.91; 1.05)
Female	Negative	1.12 (0.89; 1.40)	1.11 (0.90; 1.37)	1.04 (0.98; 1.11)	1.01 (0.96; 1.07)
**Chronic condition**					
No	None or minimal	Ref.	Ref.	Ref.	Ref.
Yes	Positive	1.23 (0.92; 1.63)	1.20 (0.89; 1.62)	1.03 (0.95; 1.12)	1.00 (0.93; 1.08)
Yes	Negative	1.00 (0.79; 1.25)	1.00 (0.80; 1.25)	1.02 (0.96; 1.09)	1.01 (0.95; 1.07)
**Household financial situation**					
Very good	None or minimal	Ref.	Ref.	Ref.	Ref.
Good	Positive	0.79 (0.56; 1.12)	0.84 (0.58; 1.20)	1.04 (0.95; 1.15)	1.03 (0.94; 1.13)
Good	Negative	0.73 (0.54; 0.97)*	0.77 (0.57; 1.04)°	1.03 (0.96; 1.11)	1.02 (0.96; 1.09)
Average to poor	Positive	0.91 (0.62; 1.36)	0.91 (0.61; 1.34)	1.08 (0.97; 1.19)	1.04 (0.95; 1.14)
Average to poor	Negative	0.57 (0.40; 0.81)*	0.64 (0.46; 0.89)*	1.07 (0.98; 1.17)	1.05 (0.97; 1.13)
**Parents' highest education**					
University	None or minimal	Ref.	Ref.	Ref.	Ref.
Lower than university	Positive	0.83 (0.56; 1.23)	0.90 (0.63; 1.29)	0.96 (0.86; 1.07)	0.98 (0.88; 1.08)
Lower than university	Negative	0.84 (0.59; 1.18)	0.79 (0.58; 1.08)	0.95 (0.87; 1.05)	1.00 (0.93; 1.07)
**Parent-child relationship**					
Very good to good	None or minimal	Ref.	Ref.	Ref.	Ref.
Less than good	Positive	1.17 (0.84; 1.63)	1.10 (0.80; 1.52)	1.03 (0.94; 1.13)	1.01 (0.92; 1.10)
Less than good	Negative	0.83 (0.66; 1.05)	0.84 (0.67; 1.05)	1.04 (0.97; 1.10)	1.03 (0.97; 1.09)
**Parents' mood**					
Very good to good	None or minimal	Ref.	Ref.	Ref.	Ref.
Average to poor	Positive	1.16 (0.76; 1.76)	1.21 (0.80; 1.82)	0.99 (0.89; 1.09)	0.98 (0.89; 1.08)
Average to poor	Negative	0.76 (0.58; 1.00)°	0.83 (0.64; 1.08)	1.06 (0.99; 1.14)	1.03 (0.97; 1.10)
**Number of friends**					
Several	None or minimal	Ref.	Ref.	Ref.	Ref.
One or less	Positive	1.00 (0.70; 1.44)	1.08 (0.75; 1.53)	0.94 (0.84; 1.05)	0.97 (0.88; 1.07)
One or less	Negative	1.02 (0.78; 1.34)	1.09 (0.84; 1.42)	0.94 (0.88; 1.01)°	0.97 (0.91; 1.03)
**Extracurricular activities**					
Yes	None or minimal	Ref.	Ref.	Ref.	Ref.
No	Positive	0.75 (0.49; 1.13)	0.79 (0.53; 1.18)	0.94 (0.84; 1.05)	0.98 (0.88; 1.09)
No	Negative	1.11 (0.81; 1.52)	1.05 (0.77; 1.42)	0.99 (0.91; 1.07)	0.99 (0.92; 1.06)
**Screen time**					
Meeting recommendations	None or minimal	Ref.	Ref.	Ref.	Ref.
Not meeting recommendations	Positive	0.92 (0.65; 1.29)	0.94 (0.68; 1.29)	1.04 (0.94; 1.15)	1.03 (0.94; 1.13)
Not meeting recommendations	Negative	1.02 (0.79; 1.31)	1.01 (0.79; 1.28)	0.99 (0.93; 1.06)	0.99 (0.93; 1.06)
**Physical activity**					
Meeting recommendations	None or minimal	Ref.	Ref.	Ref.	Ref.
Not meeting recommendations	Positive	0.93 (0.69; 1.25)	1.00 (0.74; 1.36)	0.94 (0.86; 1.02)	0.96 (0.88; 1.05)
Not meeting recommendations	Negative	0.94 (0.74; 1.19)	0.99 (0.78; 1.26)	1.00 (0.94; 1.07)	0.99 (0.94; 1.05)
**Sleep duration**					
Meeting recommendations	None or minimal	Ref.	Ref.	Ref.	Ref.
Not meeting recommendations	Positive	1.33 (0.93; 1.89)	1.20 (0.84; 1.71)	0.96 (0.86; 1.07)	0.96 (0.87; 1.06)
Not meeting recommendations	Negative	1.05 (0.81; 1.37)	0.95 (0.75; 1.20)	0.95 (0.89; 1.02)	0.98 (0.91; 1.04)

° p-value < 0.1; * p-value < 0.05. Results are incidence rate ratio (IRR) and 95% confidence intervals (CI) from generalized mixed effects models adjusted with fixed effects for age, sex, chronic condition, and household financial situation, and random effects at the child and household levels.

Table 7. Yearly incidence rate ratio of mental health difficulties according to the child COVID-19 pandemic impact with various delta constants in children aged 2-17 years old, between 2022 and 2025 (n=1907).

Type of child impact	Delta	IRR (95% CI)
None/minimal	0	Ref.
Positive impact	0	1.02 (0.98-1.06)
Negative impact	0	0.98 (0.95-1.01)
None/minimal	1	Ref.
Positive impact	1	1.02 (0.98-1.06)
Negative impact	1	0.97 (0.94-1.00)*
None/minimal	2	Ref.
Positive impact	2	1.02 (0.98-1.05)
Negative impact	2	0.96 (0.94-0.99)*

Results are incidence rate ratio (IRR) and 95% confidence intervals (CI) of pandemic impact × time interactions from generalized mixed effects models with fixed effects for age, sex, chronic condition, and household financial situation, and random effects at the child and household levels. Variable missingness is addressed with multiple imputation by chained equations with various delta constants added to the imputed SDQ total score as a sensitivity analysis.

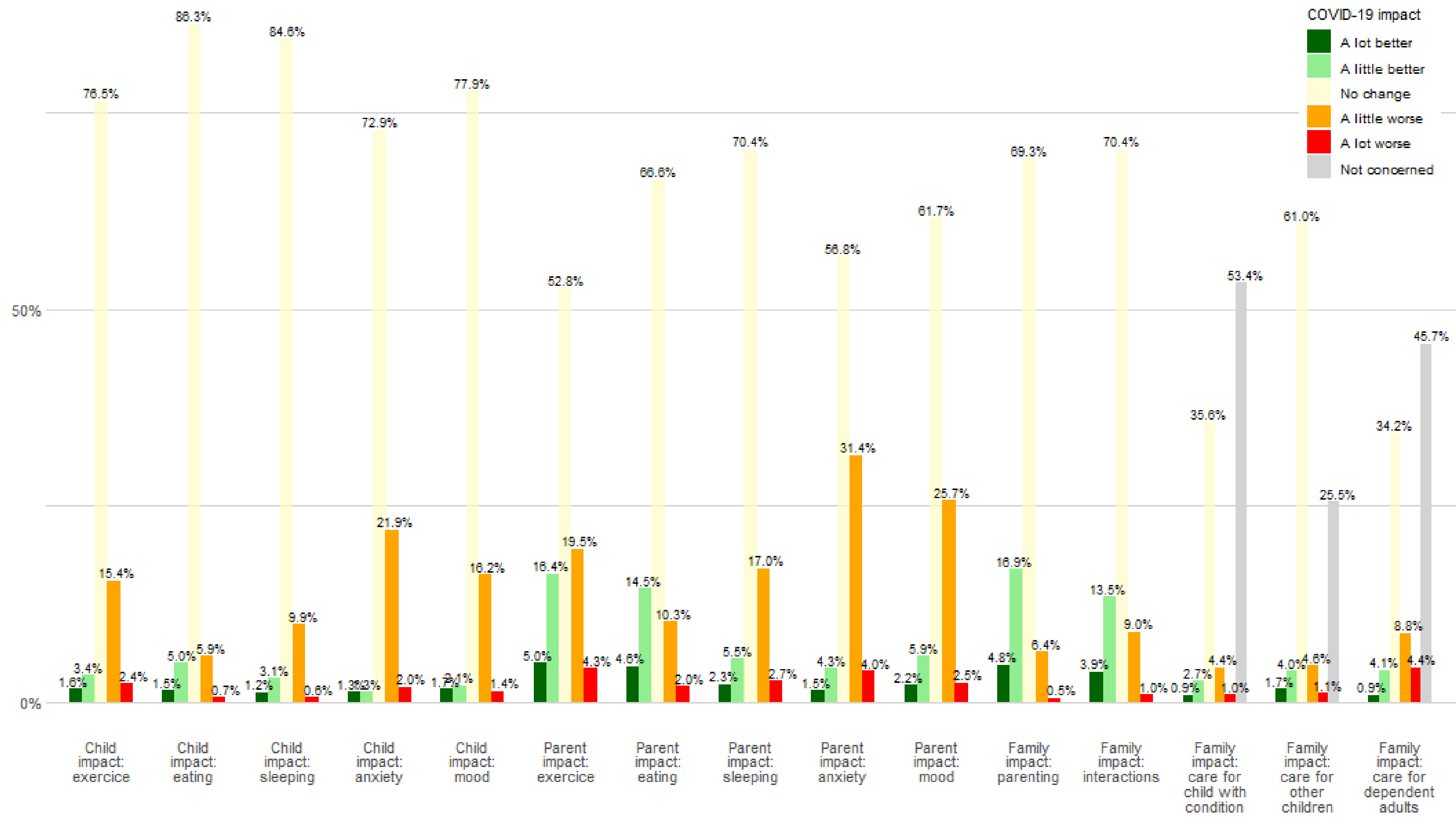

Additional figure 1. Item response to the COVID-19 Exposure and Family Impact Scales (CEFIS) at the child, parent and family level

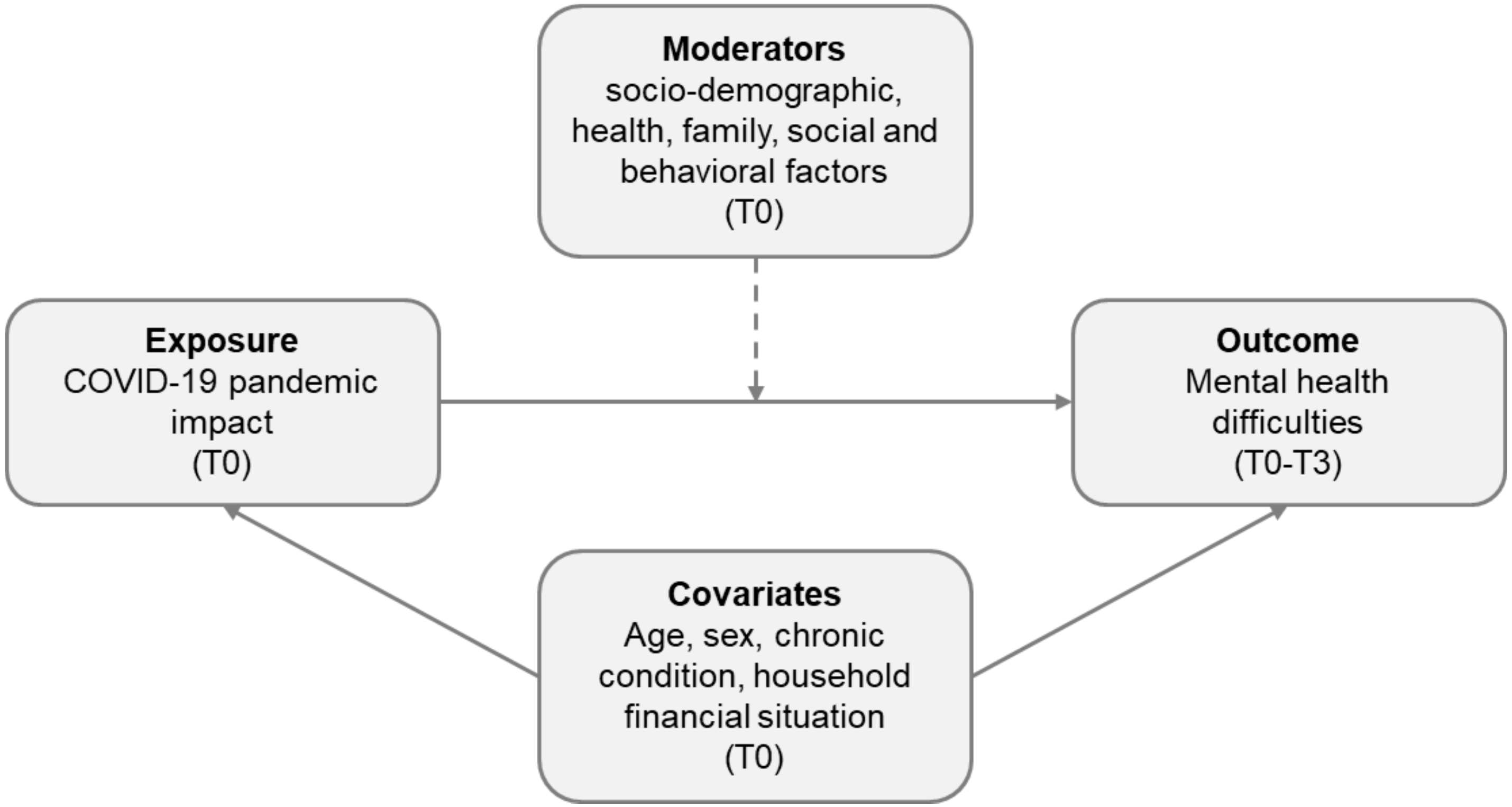

Additional figure 2. Directed acyclic graph of the hypothesized relationships underlying the main study model (plain line) and moderator's models (dashed line). T0 assessments were conducted in 2022, whether in the baseline survey for the mental health and covariates or in a thematic survey for the pandemic impact. T1-T3 assessments were conducted in May-July 2023, 2024, and 2025, respectively.

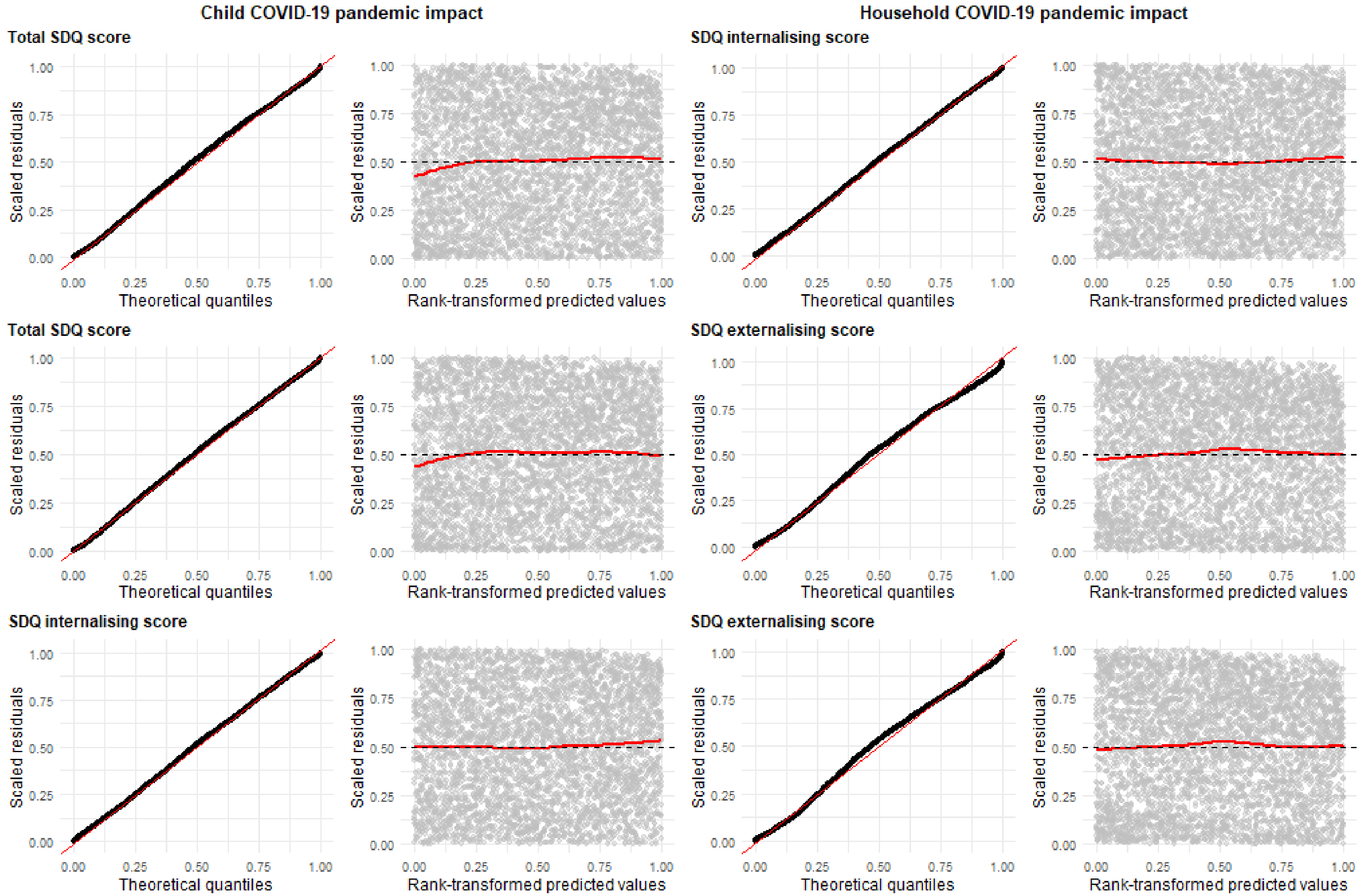

Additional figure 3. Diagnostic plots of models assessing the association between the child pandemic impact and mental health difficulties over time. Results are from generalized mixed effects models following a generalized Poisson distribution with fixed effects for age, sex, chronic condition, and household financial situation, and random effects at the child and household levels

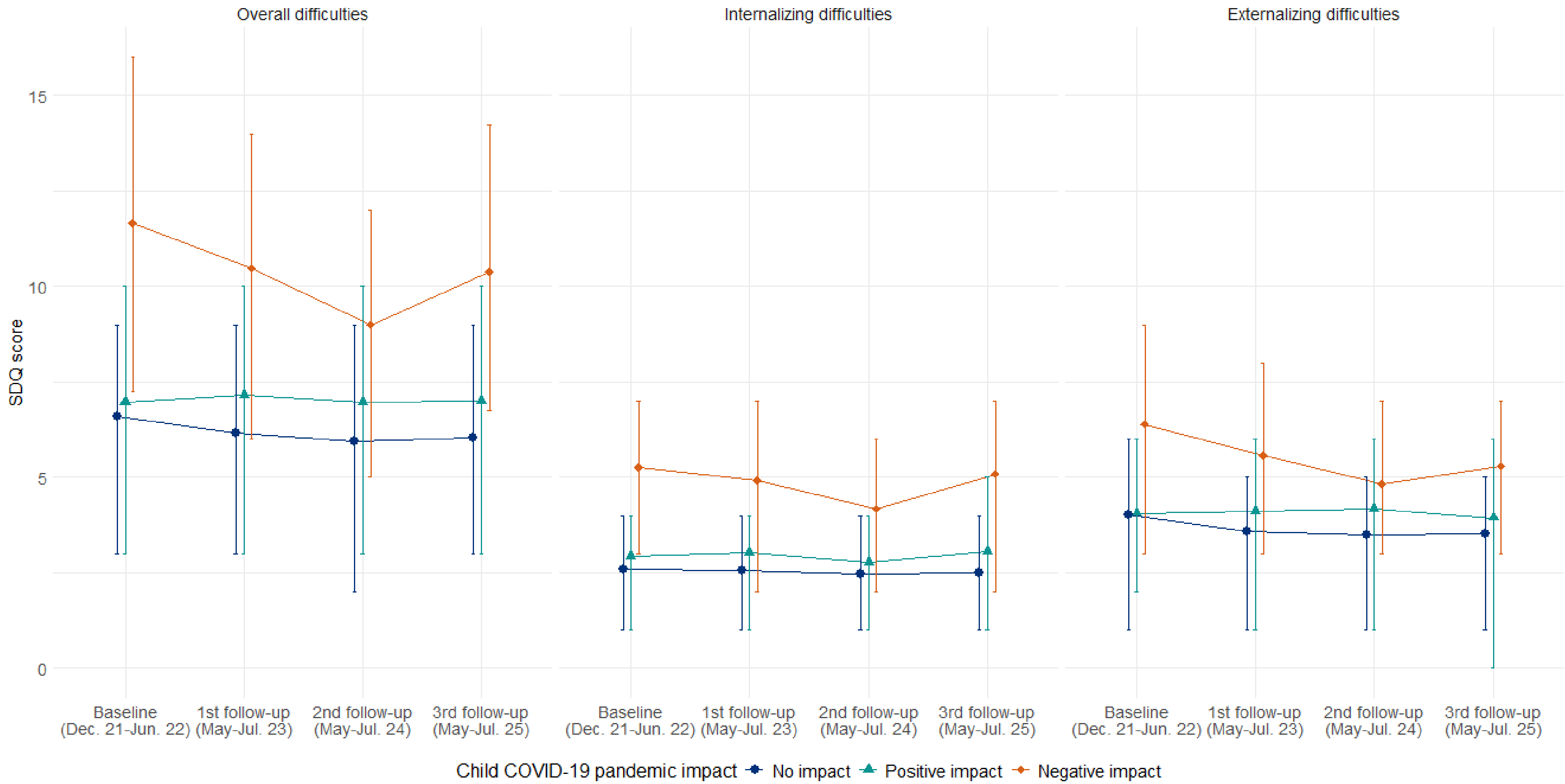

Additional figure 4. Median and interquartile range of the strengths and difficulties questionnaire (SDQ) scores across timepoints by child COVID-19 pandemic impact (n=1488).
